# Career challenges of young oncologists in Romania: a nationwide survey

**DOI:** 10.1186/s12909-026-09006-z

**Published:** 2026-03-20

**Authors:** Daniel Sur, Cristina Lungulescu-Berisha, Adelina Silvana Gheorghe, Cristian Virgil Lungulescu, Stefania Andreea Toma, Eliza Maria Armeanu, Raluca Ioana Mihaila, Elena Adriana Iovanescu, Irina Mihaela Croitoru-Cazacu, Vlad Mihai Croitoru, Radu Dragomir, Amir Hubeishie, Vlad Lupu, Silvia Mosoiu, Daniel Dulf, Teodora Alexa-Stratulat, Simona Ruxandra Volovăț, Dana Lucia Stanculeanu, Mihai Vasile Marinca, Amedeia Nita, Laurentia Gales, Mircea Dragos Median, Adina Croitoru, Serban Negru, Laura Mazilu, Razvan-Ovidiu Curca, Michael Schenker, Vlad Adrian Afrasanie

**Affiliations:** 1 Department of Medical Oncology, Iuliu Hațieganu University of Medicine and Pharmacy, Cluj-Napoca, 400012 Romania; 2 Department of Medical Oncology, The Oncology Institute Prof. Dr. Ion Chiricuţă, Cluj-Napoca, 3400 Romania; 3 Department of Biochemistry, University of Medicine and Pharmacy of Craiova, Craiova, 200349 Romania; 4 Department of Medical Oncology, Institute of Oncology ”Prof. Dr. Al. Trestioreanu” Bucharest, Bucharest, 022328 Romania; 5 Department of Oncology, University of Medicine and Pharmacy of Craiova, Craiova, 200349 Romania; 6 Department of Medical Oncology, Ponderas Academic Hospital, Bucharest, 014142 Romania; 7 Grigore T. Popa University of Medicine and Pharmacy, Iasi, 700115 Romania; 8 Department of Oncology, Carol Davila University of Medicine and Pharmacy, Bucharest, 050474 Romania; 9 Department of Oncology, Fundeni Clinical Institute, Bucharest, 022328 Romania; 10 Oncology Department, Oncohelp Oncology Center, Timisoara, 300425 Romania; 11 Department of Oncology, Victor Babes University of Medicine and Pharmacy, Timisoara, 300041 Romania; 12 Spitalul Clinic Municipal Cluj-Napoca, Cluj-Napoca, Romania; 13 Department of Medical Oncology-Radiation Therapy, Faculty of Medicine, Grigore T. Popa University of Medicine and Pharmacy, Iasi, Romania; 14 Department of Medical Oncology, Regional Institute of Oncology, Iasi, Romania; 15 Department of Medical Oncology, Hospital City Ploiesti, Ploiești, Romania; 16 Donna Oncology, Filantropia Clinical Hospital, Bucharest, Romania; 17 Ovidius University of Constanta, Constanta, Romania; 18 Department of Medical Oncology, Elysee Hospital Alba Iulia, Alba, Romania

**Keywords:** Survey, Romania, Workforce development, Young oncologists, Training, Mentorship, Research, Early-career physicians

## Abstract

**Background:**

Medical oncology is a young specialty in Romania, where early-career oncologists face challenges related to training, mentorship, and research. This study explored their professional status and needs to inform workforce development.

**Methods:**

An online survey was conducted between February and April 2025 among physicians under 40 years of age who were members of the Romanian National Society of Medical Oncology (SNOMR). The questionnaire (60 items) covered demographics, employment, mentorship, research, and professional development. Descriptive analysis was performed.

**Results:**

Of 456 eligible members, 169 responded (response rate: 37%). Most were residents based in oncology institutes or university hospitals. Job insecurity was common, with 57% on temporary contracts. Nearly all respondents (99%) had clinical duties, but only 21% engaged in research and 9% in teaching. Mentorship was inconsistently available (61% had a mentor), yet interest in structured programs was very high (88% for national and 87% for international initiatives). Research involvement was limited: 46% reported no activity, 85% lacked funding, and only 28% published in the past year. While over 80% expressed interest in fellowships or exchanges, only 16% had trained abroad. Most (85%) were ESMO members, but only half considered SNOMR support sufficient.

**Conclusion:**

Young oncologists in Romania are motivated but face significant barriers, including job instability, insufficient mentorship, and limited research opportunities. Expanding structured mentorship, ensuring protected research time, and strengthening international mobility are essential. Recent SNOMR initiatives represent important first steps toward addressing these gaps.

**Supplementary Information:**

The online version contains supplementary material available at 10.1186/s12909-026-09006-z.

## Background

Medical oncology is a relatively young specialty that emerged in the 1960s. Major scientific societies, such as the European Society for Medical Oncology (ESMO) and the American Society of Clinical Oncology (ASCO), have played a pivotal role in improving the quality of training, although the level of development has varied across countries [1]. Engaging well-trained and highly motivated, early-career oncologists is essential to sustain the advancement of the field and ensure the delivery of high-quality care to patients with cancer [2]. Notably, oncologists aged 40 or younger constitute a significant proportion of the profession; in 2016, 40% of ESMO members fell into this category [3].

Romania, like other Eastern European countries, continues to face structural disparities compared to Western Europe, particularly in healthcare and education, although three decades of post-communist transformations have gradually improved health system performance and population health outcomes across the region [4]. Within the medical field, oncology exemplifies these challenges [5]. Medical oncology was only recently recognized as a distinct specialty in Romania, formally added to the national list of specialties in 2011 [6]. According to the OECD 2025 report, Romania faces a significant shortage of oncologists, with only 4.4 oncologists per 100,000 inhabitants in 2022, compared to an EU average of 13.7 [7]. These limitations have constrained both the number of trained specialists and the development of robust professional pathways, leaving the field underprepared to meet the growing national cancer burden [6].

Over the past 15 years, rising cancer incidence has prompted health authorities to expand oncology training programs, resulting in a rapid increase in residents and specialists number [8–12]. In parallel, the Romanian Society of Medical Oncology (SNOMR) has evolved into an active professional body, while economic improvements have created conditions more favorable for professional development [4, 13].

Despite these advances, significant gaps remain between Eastern and Western Europe in training quality, job stability, research opportunities, and access to cutting-edge clinical practices. Eastern European oncologists consistently report lower job satisfaction, likely reflecting healthcare infrastructure disparities [14]. Young oncologists from middle-income countries in particular face barriers to international collaboration, structured mentorship, research facilities, and career advancement. Addressing these inequities is critical for both professional development and improved cancer outcomes nationwide [15].

To better understand these challenges and inform targeted interventions, the Romanian Young Oncologists Group, with the support of SNOMR, conducted a nationwide survey. This study aims to identify the professional challenges faced by early-career medical oncologists in Romania and to outline feasible strategies for aligning their training, employment stability, and career development with European standards.

The principal objective of the survey was to describe the professional landscape and the career-related challenges of young medical oncologists in Romania. More precisely, our objectives were to: (1) outline their professional and sociodemographic traits; (2) assess their access to research, training, and mentorship; and (3) identify and describe the main perceived obstacles to career advancement, such as workload, work-life balance, and migration ambitions.

## Methods

A multidisciplinary team of early-career oncologists from the Romanian Young Oncologists Group, with guidance from a senior SNOMR-affiliated mentor, developed a questionnaire. The survey was distributed online via Google Forms between February 1 and April 1, 2025, to all medical oncology physicians under 40 years of age, in line with the ESMO definition of young oncologists. The survey was distributed exclusively only to active members of the Romanian National Society of Medical Oncology (SNOMR). Participation was voluntary and anonymous. Of 456 eligible SNOMR members, 169 completed the survey, yielding a response rate of 37%.

In order to create the survey, members of the Romanian Young Oncologists Group reviewed the literature on career development and research interest of early-career oncologists. They also looked at pertinent survey instruments from previous studies conducted by ESMO and other international oncology societies. The final variant of the questions was created to address institutional contexts and training paths unique to Romania. Also, topics on working conditions and job satisfaction were adapted to reflect the national environment. A multidisciplinary panel’s expert evaluation proved content validity by assessing each item for comprehensiveness, clarity, and relevance; minor language changes resulted. After that, minor adjustments were made to the item language and question order after the questionnaire was pilot-tested in a convenience sample of 8–10 young oncologists from different training levels and institutions.

The final version of the questionnaire consisted of 60 items (see Supplementary File 1), including structured closed-ended questions (single-choice, multiple-choice, dichotomous yes/no items, Likert-type items, and numerical response questions), eight primary open-ended questions, and several optional free-text “Other” response fields. The open-ended responses were reviewed by members of the study team and grouped into thematic categories through descriptive content categorization in order to identify recurrent themes and support interpretation of the quantitative findings.

Given the exploratory and primarily descriptive design of the study, no formal qualitative methodology was applied. Any observed differences between subgroups are presented as descriptive only and were not tested for statistical significance.

We assessed the professional status of young medical oncologists in Romania across six key domains: (1) demographic and professional background (2), employment and job stability (3), mentorship and professional development (4), research and academic engagement, and (5) professional development initiatives.

## Results

### Demographic and professional background

**Fig. 1:**
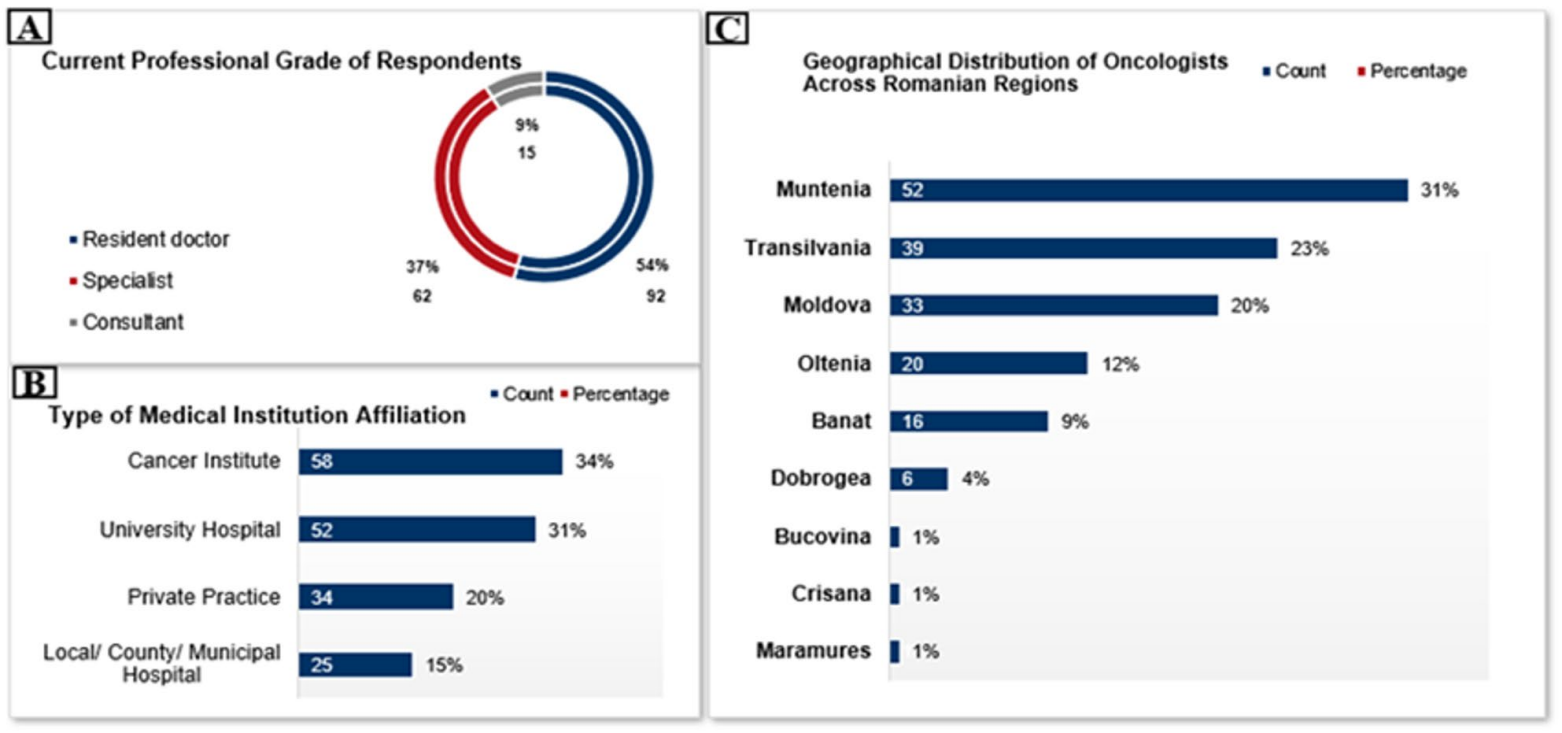
Demographic and professional background: current professional grade of respondents (**A**), type of medical institution affiliation (**B**) and geographical distribution of oncologists across Romanian regions (**C**)

The survey demonstrated a predominance of resident doctors, who constituted 54% (*n* = 91) of respondents, followed by specialists, who made up 37% (*n* = 63), and consultants, who represented 9% (*n* = 15) (Fig. 1a). Regarding workplace affiliation, the majority of young oncologists in Romania were employed at Cancer Institutes 34% (*n* = 57) or University Hospitals 31% (*n* = 52), while 20% (*n* = 34) worked in private practices and 15% (*n* = 26) were affiliated with Local, County, or Municipal Hospitals (Fig. 1b). The geographic distribution of respondents revealed significant regional differences, with the largest proportion practicing in Muntenia 31% (*n* = 52), followed by Transilvania 23% (*n* = 39) and Moldova 20% (*n* = 34). Oltenia accounted for 12% (*n* = 20), Banat for 9% (*n* = 15), and Dobrogea for 4% (*n* = 7), while Bucovina, Crișana, and Maramureș each represented approximately 1% (*n* ≈ 2) of respondents (Fig. 1c).

### Employment and job stability

**Fig. 2:**
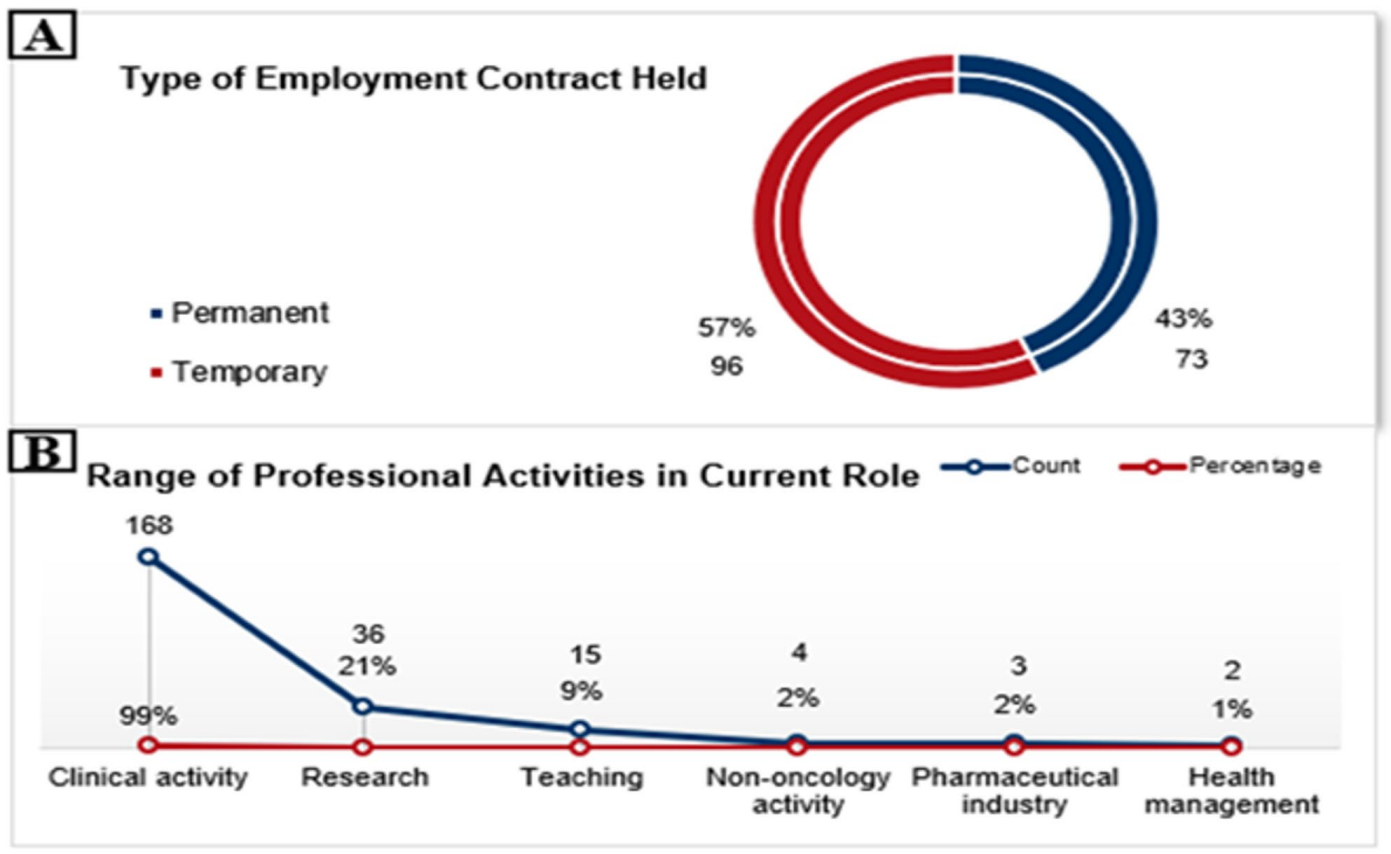
Employment and job stability: type of employment contract held (**A**), range of professional activities in current role (**B**) from the 169 responders a percentage of 99% had clinical activities, a percentage of 21% from the 169 responders had also research activities; 9% were involved in teaching besides other roles; respectively 2% had non-oncology and pharmacology industry roles besides clinical activities or research; only 1% was involved in health management

Among respondents (*n* = 169), 43% (*n* = 73) held permanent contracts, while 57% (*n* = 96) were employed under temporary agreements (Fig. 2a). Almost all participants (99%, *n* = 167) reported engagement in clinical work. In contrast, involvement in other professional domains was limited: 21% (*n* = 36) participated in research, 9% (*n* = 15) in teaching, and fewer than 2% (*n* = 3) reported activity in the pharmaceutical industry or healthcare management (Fig. 2b).

### Mentorship and professional development

**Fig. 3:**
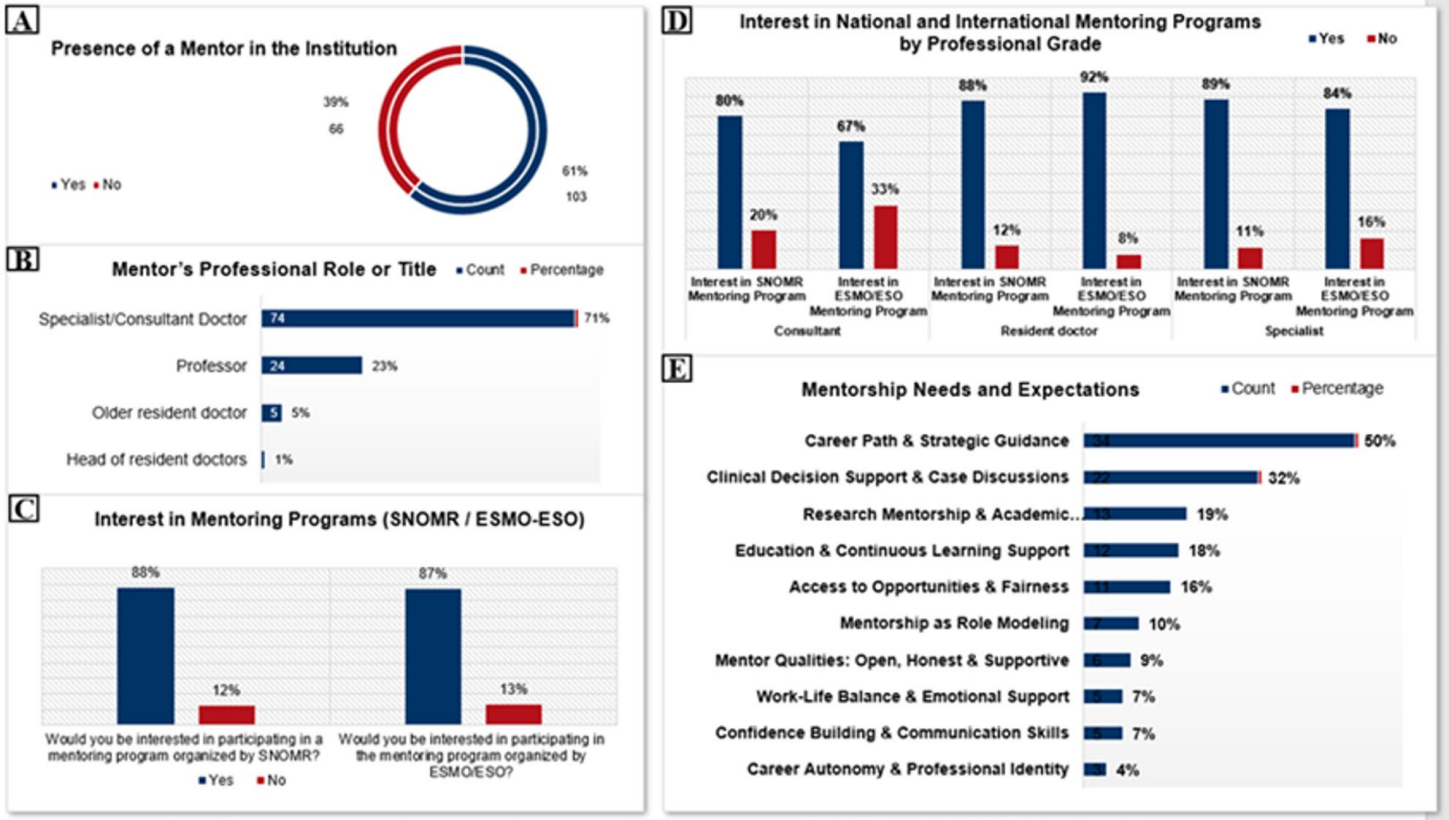
Mentorship and professional development: presence of mentor in the institution **A**, mentor’s professional role **B**, interest in mentoring programs (SNOMR/ESMO-ESO) **C**, interest in national and international programs by professional grade **D**, mentorship needs and expectations **E**

A majority of young oncologists (61%, *n* = 103) reported having a mentor within their institution, while 39% (*n* = 66) indicated the absence of such support (Fig. 3a). Among those with mentors, most identified their mentor as a specialist or consultant doctor (71%, *n* ≈ 73), followed by professors (23%, *n* ≈ 24). Fewer respondents reported senior residents (5%, *n* ≈ 5) or heads of resident doctors (1%, *n* ≈ 1) as mentors (Fig. 3a).

Nearly all respondents expressed interest in formal mentoring programs: 88% (*n* = 149) were willing to participate in initiatives organized by SNOMR, and 87% (*n* = 147) supported mentorship programs offered by international organizations such as ESMO/ESO (Fig. 3c). Interest varied slightly by professional grade, with residents showing the highest engagement (88%, *n* ≈ 80 for SNOMR; 92%, *n* ≈ 84 for ESMO/ESO), specialists reporting similarly strong interest (89%, *n* ≈ 56 and 84%, *n* ≈ 53, respectively), and consultants demonstrating somewhat lower—though still substantial—enthusiasm (80%, *n* ≈ 12 for SNOMR and 67%, *n* ≈ 10 for ESMO/ESO) (Fig. 3d). When asked about specific mentorship needs, respondents most frequently prioritized guidance on career path and strategic planning (50%, *n* ≈ 85). Other areas included clinical decision-making and case discussions (32%, *n* ≈ 54), research and academic development (19%, *n* ≈ 32), education and continuous learning (18%, *n* ≈ 30), and equitable access to opportunities (16%, *n* ≈ 27). Less commonly cited needs included role modeling (10%, *n* ≈ 17), emotional support and work–life balance (7%, *n* ≈ 12), and confidence-building (7%, *n* ≈ 12) (Fig. 3e).

**Fig. 4:**
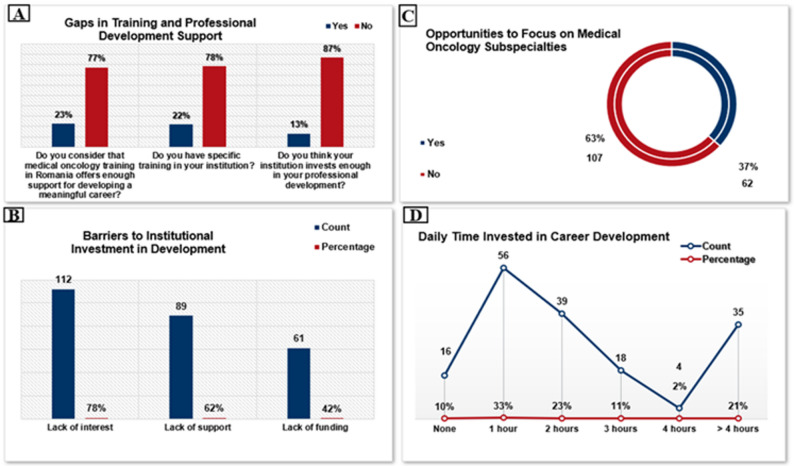
Mentorship and professional development: gaps in training and professional development support **A**, barriers to institutional investment in development **B**, opportunities to focus on medical oncology subspecialties **C**, daily time invested in career development **D**

Only 23% of respondents (*n* = 39) felt that medical oncology training in Romania provides sufficient support for building a meaningful career. Similarly, 22% (*n* = 37) reported access to structured training within their institution, and just 13% (*n* = 22) believed that their institutions actively invest in their professional development (Fig. 4a).

Among those who did not perceive adequate institutional support, the most frequently cited reasons were lack of institutional interest (78%), insufficient support mechanisms (62%), and inadequate funding (42%) (Fig. 4b).

Most young oncologists (63%, *n* = 107) reported not having the opportunity to subspecialize or focus on a specific tumor type, while only 37% (*n* = 62) were currently engaged in subspecialized practice (Fig. 4c).

Regarding daily time allocated to career development, one-third of respondents (33%, *n* = 56) reported dedicating one hour per day, 23% (*n* = 39) two hours, and 11% (*n* = 19) three hours. Notably, 21% (*n* = 35) invested more than four hours daily, whereas 10% (*n* = 17) reported having no time for professional development (Fig. 4d).

### Research and academic engagement

**Fig. 5:**
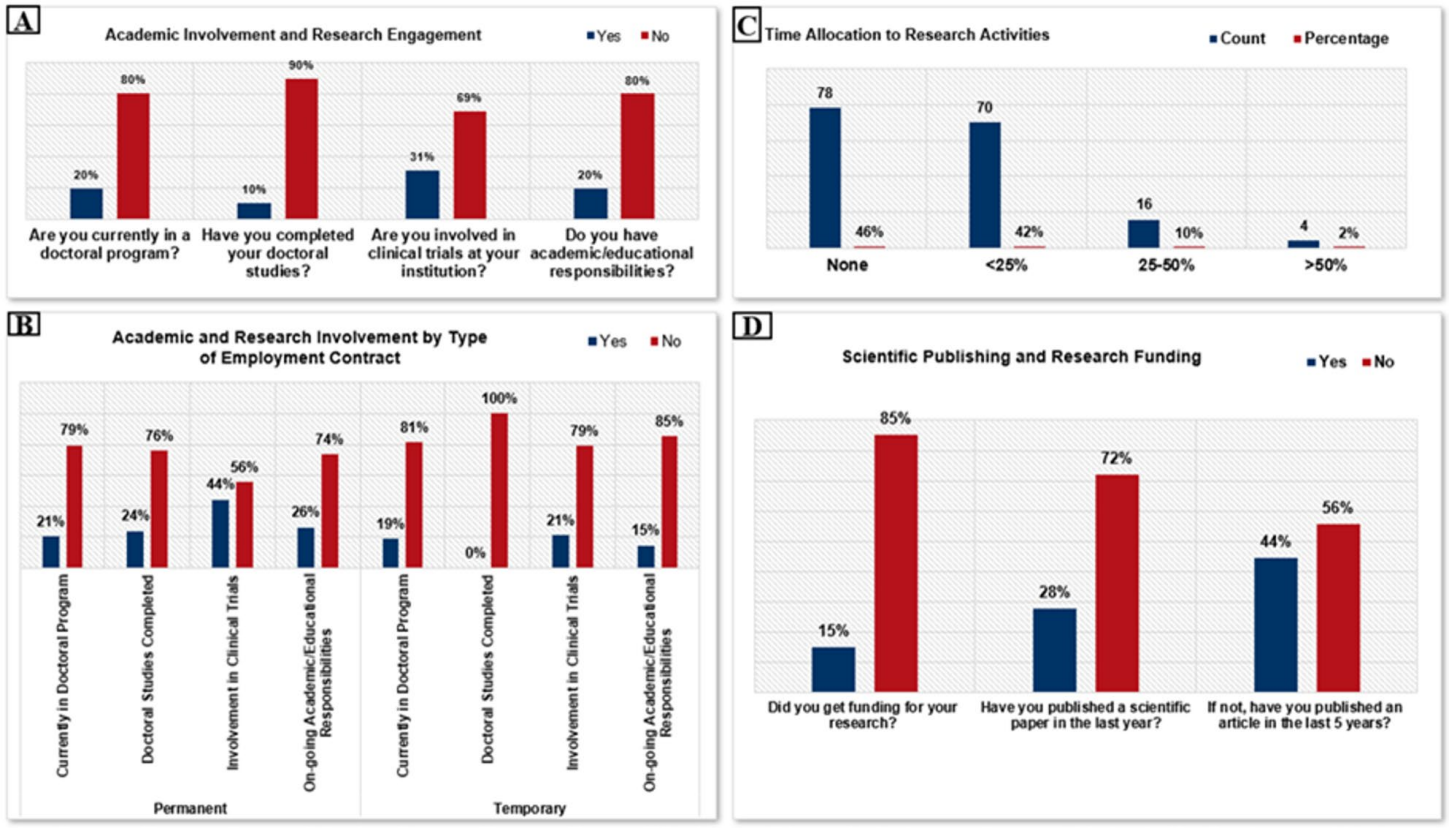
Research and academic engagement: Academic involvement and research engagement **A**, Academic and research involvement by type of employment contract **B**, time allocation in research activities **C**, scientific publishing and research funding **D**

Survey results reveal low engagement in structured academic development among young oncologists in Romania. Only 20% (*n* = 34) were currently enrolled in a doctoral program, while 10% (*n* = 17) had completed one. Additionally, just 20% (*n* = 34) reported academic or educational responsibilities. Research involvement through clinical trials was also limited, with only 31% (*n* = 52) participating in such activities (Fig. 5a). Employment status appeared to influence academic engagement, as among respondents with permanent contracts, 44% (*n* ≈ 41) were involved in clinical trials and 26% (*n* ≈ 24) had academic duties, compared to 21% (*n* ≈ 11) and 15% (*n* ≈ 8), respectively, among those with temporary contracts. Furthermore, 24% (*n* ≈ 22) of permanently employed respondents had completed doctoral studies, whereas none of those on temporary contracts had done so (Fig. 5b).

Overall, research activity among young oncologists in Romania remained limited. Nearly half (46%, *n* = 78) reported no involvement in research, while 42% (*n* ≈ 71) dedicated less than 25% of their time to such work. Only 12% (*n* = 20) allocated more than 25% of their time to research, indicating that for most, research plays little or no role in daily responsibilities (Fig. 5c). Financial support for research was also scarce: only 15% (*n* = 25) of respondents reported receiving research funding, while the vast majority (85%, *n* = 144) lacked any financial support (Fig. 5d).

**Fig. 6:**
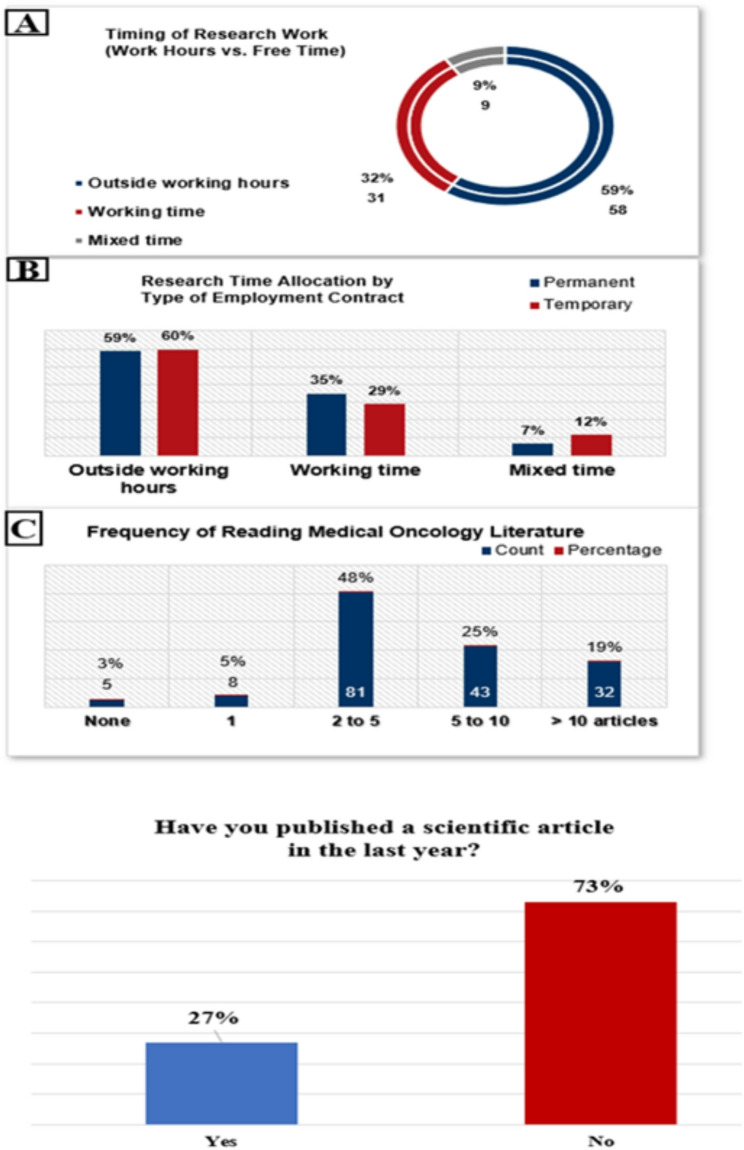
Research and Academic Engagement: Timing of Research Work (Work hours vs. Free Time) **A**, Research Time Allocation by Type of Employment Contract **B**, Frequency of Reading Medical Oncology Literature **C**, Scientific article publication in the last year **D**

Among respondents engaged in research, 59% (*n* ≈ 100) conducted it outside official working hours, 32% (*n* ≈ 54) during working hours, and 9% (*n* ≈ 15) in a mixed capacity (Fig. 6a). Cross-tabulation by contract type showed minimal variation, as both permanent and temporary staff predominantly conducted research outside working hours (59% and 60%, respectively). Only 35% (*n* ≈ 35) of permanently employed and 29% (*n* ≈ 16) of temporarily employed oncologists reported performing research during their work hours (Fig. 6b). Engagement with medical oncology literature was moderate overall. Nearly half of respondents (48%, *n* ≈ 81) reported reading two to five articles monthly, while 25% (*n* ≈ 42) read five to ten articles. A smaller proportion (19%, *n* ≈ 32) exceeded ten articles per month, whereas 8% (*n* ≈ 14) read one or fewer (Fig. 6c). In terms of research output, 28% (*n* ≈ 47) had published a scientific paper within the past year. Among those who had not published in the last year, 44% (*n* ≈ 54) reported at least one publication within the previous five years (Fig. 6d).

### Professional development initiatives

**Fig. 7:**
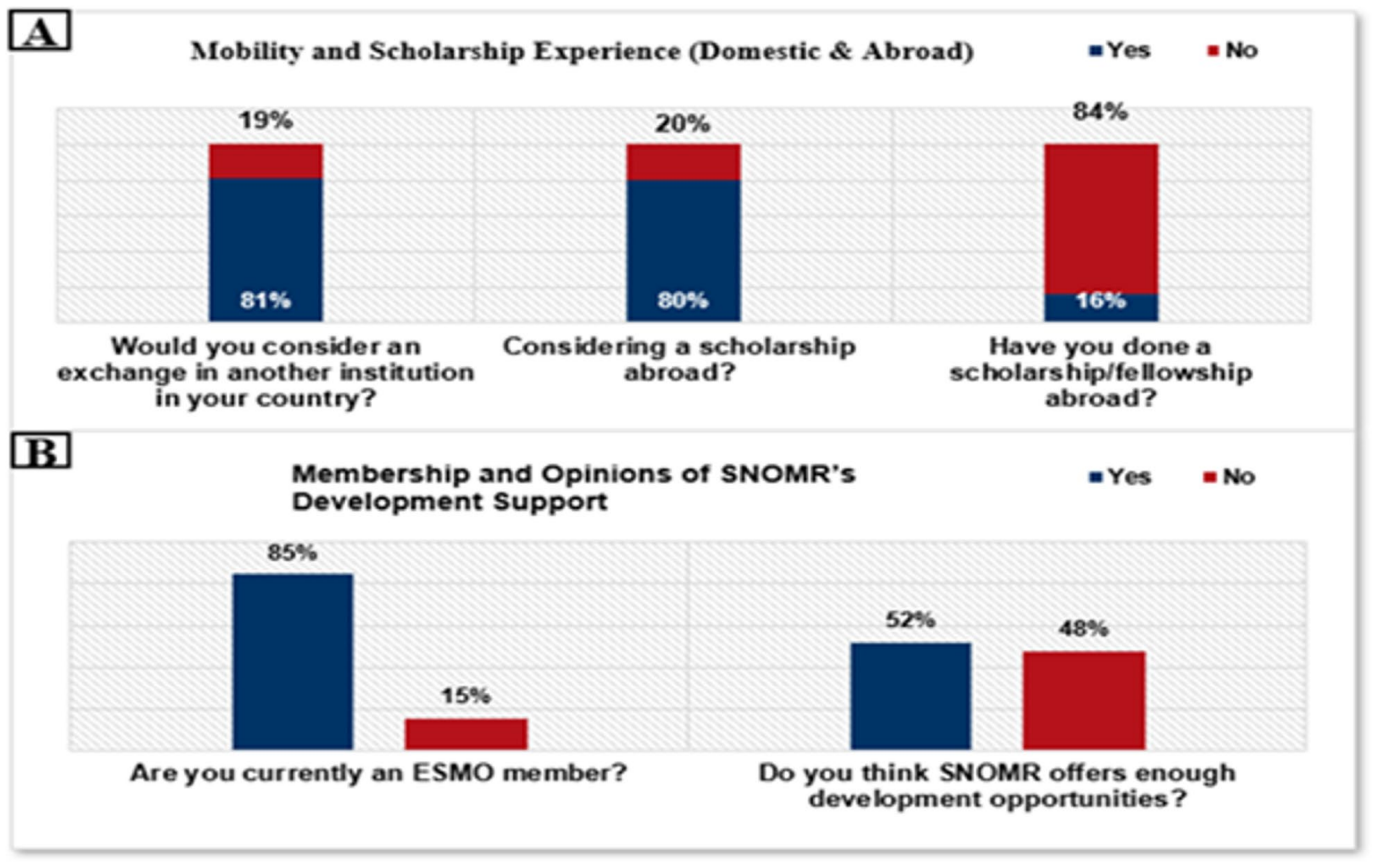
Professional development initiatives: mobility and scholarship experience **A**, Membership and Opinions of SNOMR’s Development Support **B**

The survey revealed strong interest among young oncologists in Romania in professional mobility and international exposure. A large majority indicated they would consider either a domestic institutional exchange (81%, *n* ≈ 137) or a international fellowship (80%, *n* ≈ 135). However, only 16% (*n* ≈ 27) had actually completed a fellowship or scholarship outside the country (Fig. 7a). Most respondents (85%, *n* ≈ 144) reported membership in the European Society for Medical Oncology (ESMO). In contrast, perceptions of national support through SNOMR were more divided: 52% (*n* ≈ 88) felt that SNOMR provided sufficient professional development opportunities, while 48% (*n* ≈ 81) disagreed (Fig. 7b).

**Fig. 8:**
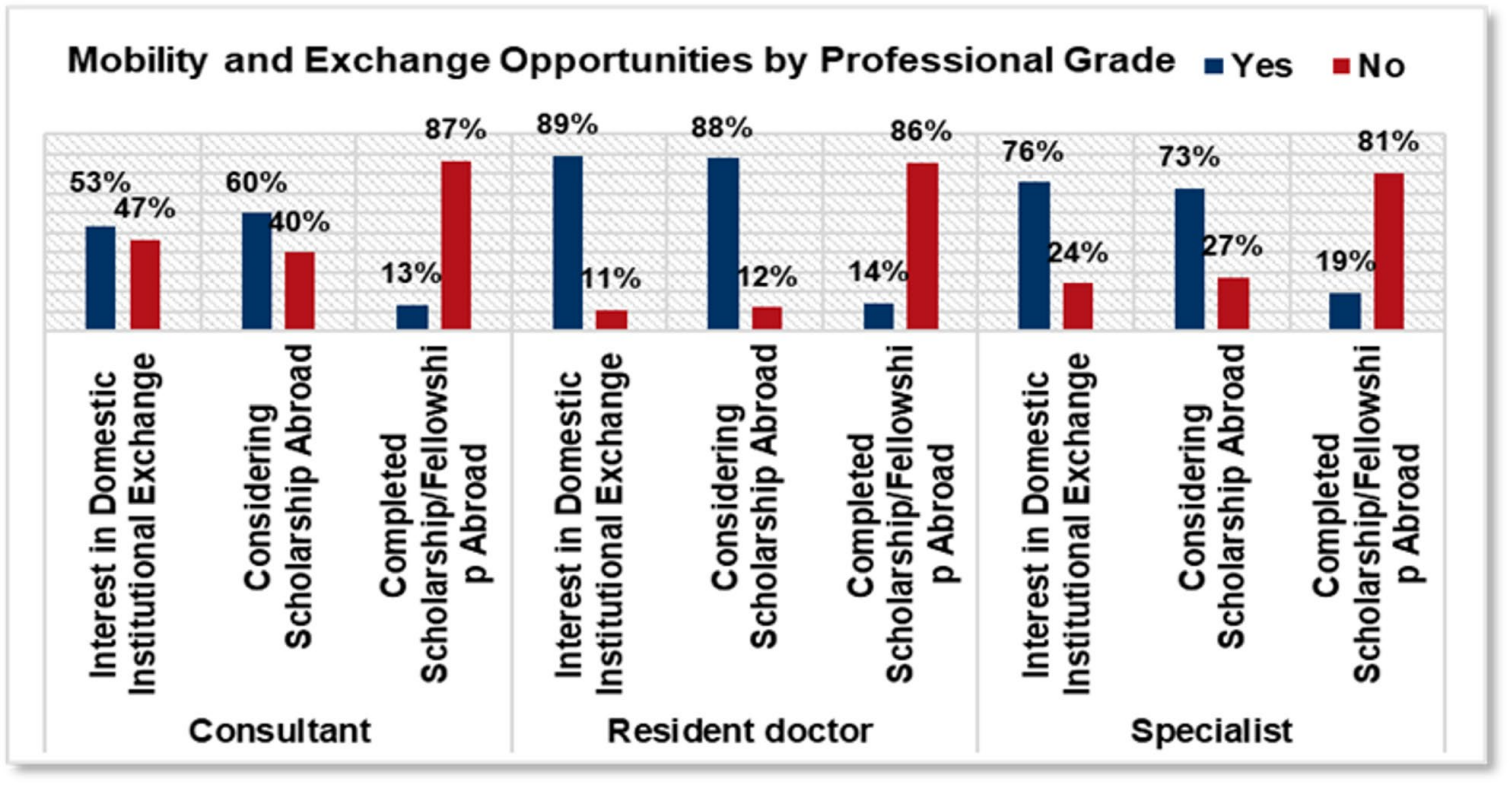
Mobility and exchange opportunities by professional grade

Interest in both domestic and international programs was highest among resident doctors, with 89% (*n* ≈ 81) open to domestic exchanges and 88% (*n* ≈ 80) considering scholarships abroad. Specialists also demonstrated substantial interest, with 76% (*n* ≈ 48) supporting domestic exchanges and 73% (*n* ≈ 46) considering international scholarships. Consultants reported the lowest engagement, with 53% (*n* ≈ 8) open to local exchanges and 60% (*n* ≈ 9) to international opportunities. Completed fellowship experience was slightly more common among specialists (19%, *n* ≈ 12) compared with residents (14%, *n* ≈ 13) and consultants (13%, *n* ≈ 2) (Fig. 8).

## Discussion

### Demographic and professional background

Our study revealed that the majority of survey respondents were young oncologists in residency training, followed by a smaller proportion of consultants. This finding highlights the strong interest in professional development among physicians at the beginning of their careers. As residency training centers in Romania are mainly represented by oncology institutes and university hospitals, nearly two-thirds of respondents reported working in such institutions, with substantially fewer employed in county hospitals or private facilities. This distribution reflects both the concentration of medical personnel in major training centers and the relative shortage of young oncologists in smaller hospitals, underscoring the current residency training policy and the unequal workforce distribution. Regional representation of respondents mirrored the actual density of oncologists, with the highest participation observed in Muntenia, Transilvania, and Moldova, where the largest number of specialists are currently located. This is not surprising because in nearly half of Romania’s counties, the density of specialized oncologists and oncology residents remains critically low, with 19 counties having fewer than 2 per 100,000 population and 15 counties ranging between 2 and 4 per 100,000 [6]. This type of pattern was similar in a survey conducted in Spanish young oncologists that highlighted that most participants are in Catalonia, Madrid and Valencia, the regions with the most developed university hospitals [16]. To reduce the uneven distribution of young oncologists in Romania, satellite training centers in county hospitals with rotations from major institutes could broaden clinical exposure. Mentorship and teleoncology programs would support career development. Coordinated national workforce planning would ensure a more equitable allocation of oncology services.

### Employment and job stability

The survey highlights a pronounced job instability among young oncologists in Romania, primarily due to the high proportion of residents, 54% of respondents were unable to hold permanent positions, unlike specialists and consultants. This situation is better than in Spain, where only 16% of the surveyed young oncologists reported having a permanent employment contract [17]. Nearly all respondents (99%) reported clinical duties as their main responsibility, with only 20% engaged in research and approximately 10% in teaching. This pattern reflects national health policies that prioritize clinical service over academic and research roles, despite the growing need for oncology specialists. In contrast to other European Union countries, Romania faces substantial structural challenges in creating academic and research positions with reduced clinical duties [14]. This imbalance not only limits time for research but also diminishes the international competitiveness of the Romanian oncology workforce, underscoring the need for policy reforms to support integrated clinical, research, and teaching pathways [15].

Creating integrated clinical-academic career paths with protected time for research and teaching, together with more permanent positions, could improve job stability for young oncologists in Romania. Combined with structured mentorship, these measures would support professional growth.

### Mentorship and professional development

Early-career Romanian oncologists find great meaning in their profession, yet our survey reveals critical gaps in mentorship and structured career support. Many young oncologists must make difficult career decisions on their own because, despite the fact that 61% of respondents have a mentor at their institution, a significant 39% do not have any mentorship.

In Romania, mentorship remains largely informal and hierarchical, typically provided by senior physicians and with few structured programs available. In our cohort, 88% of respondents expressed willingness to participate in a national mentorship program organized by SNOMR, with a comparable proportion interested in international opportunities, such as those offered by ESMO. This strong demand underscores the widely recognized role of mentorship in enhancing career satisfaction and professional development in oncology [3, 16]. The absence of formal mentorship is particularly concerning given the high prevalence of burnout among young oncologists, a phenomenon driven by demanding clinical workloads, emotional strain from caring for seriously ill patients, and systemic pressures within healthcare [18, 19, 3]. Such regional disparities likely reflect contextual contributors, including healthcare system organization, workload distribution, and insufficient institutional support [20]. Consistent with these findings, only 23% of Romanian respondents felt their training programs provided adequate support for career advancement, and just 13% perceived institutional investment in their professional growth, highlighting the urgent need for structured mentorship initiatives and institutional strategies to improve workforce sustainability. In Romania, the majority of young oncologists (63%) reported lacking the opportunity to pursue a specific subspecialty or tumor type within medical oncology, with only 37% currently practicing in a subspecialized field. By contrast, data from Spain indicate that nearly three-quarters (73%) of respondents had subspecialized in a primary area of interest, although without national programs clearly established, like other countries [17, 21].

It is imperative to close these gaps by putting in place institutional career-development plans and structured mentorship programs, both domestically and abroad. These programs can support career-stage-appropriate psychosocial support, clinical skill development, and strategic career planning, leading to increased job satisfaction [19].

Mentorship programs are at this moment supported by global oncology organizations, such as ESMO and ESO [2]. However, access to these programs for young oncologists is limited, as there are a finite number of spots available, and competition is high. Therefore, recognizing this need, SNOMR has launched a mentorship and fellowship program aimed at providing support for young oncologists in Romania, with much broader access. Investing in mentorship infrastructure is expected to result in a more resilient and self-assured oncology workforce, particularly given the high level of enthusiasm expressed by trainees and young specialists [13].

### Research and academic engagement

Research participation among Romanian young oncologists remains limited. Just 20% of respondents say they are enrolled in or have finished a doctoral (PhD) program, and only 20% say they have any teaching responsibilities in academia. Just 12% devote more than 25% of their professional time to research activities, and nearly half do no research at all. This low level of engagement is not wholly unexpected, as establishing a career path as a clinician-scientist is difficult without funding, protected time, and structured support—all of which seem to be in short supply in our country. Actually, 59% of people who do research do so primarily after hours and on their own time. The fact that research is not incorporated into working hours raises concerns because it implies that employers do not give enough time for academic pursuits or value research as a component of the professional development of aspiring physicians [16].

Stratified analysis (Supplementary Table S2) demonstrates progressive academic engagement and research integration across training stages, with residents exhibiting limited formal academic exposure compared to specialists and consultants. These differences may be explained by the challenges specific to each professional stage. It is well recognized that residency is primarily focused on the development of core specialist competencies and is often associated with substantial clinical workload, which may limit access to academic opportunities and research involvement.

Similar findings were reported in Spain, where only 7% of young oncologists were involved in research as part of their professional duties, with half of this activity occurring outside regular working hours [13, 22]. A recent EORTC global survey identified insufficient protected research time, limited funding, and lack of formal mentorship as key barriers to early-career oncologists’ research engagement [23]. This pattern contrasts with trends observed in the United States, where approximately 60% of medical oncologists undergoing subspecialization engage in research, highlighting a significant gap between current involvement and the interest and needs in this area [22].

The importance of formal research training and protected research time for oncology trainees has been consistently emphasized as essential for developing evidence-based practice and innovation [24]. Our findings mirror global concerns that heavy clinical workloads and job insecurity slow academic advancement [14]. Notably, Romanian respondents with permanent contracts were nearly twice as likely to engage in academic work or clinical trials compared with those on temporary contracts, suggesting that job stability provides the confidence and opportunity needed to pursue research activities, whereas precarious employment prioritizes clinical duties. Financial barriers were also evident, with 85% of participants reporting lack of research funding, a limitation strongly associated with reduced scholarly productivity. International evidence confirms that research output among young oncologists correlates positively with mentorship and grant availability [25].

In our cohort, only 28% had published within the previous 12 months, reflecting limited access to structured guidance and resources. Similar challenges have been described in middle-income countries, where restricted funding, inadequate infrastructure, limited access to literature, and poor international collaborations further constrain academic productivity [15]. An international survey reported that many young oncology investigators contribute personal, unpaid time to research, face limited access to experienced mentors—particularly in Eastern Europe—and are underrepresented in trial administration, design, and analysis, which remain dominated by senior professionals [26]. These findings suggest that many talented young oncologists remain underutilized in advancing oncology research. Importantly, this is not unique to Romania; even in more developed countries such as Spain, a substantial proportion of trainees expressed a desire to increase their research involvement highlighting the need for solutions that could improve the local context [17].

From an educational perspective, our findings reveal variability in postgraduate oncology training in Romania. Access to structured learning, mentorship, and protected research time is inconsistent, leading to differences in supervision and academic exposure. Residents are less involved in research and clinical trials and have limited early sub specialization opportunities, suggesting that advanced competency development often depends on institutional context rather than national standardized pathways. These differences reflect structural and training-stage factors, not individual capability.

High clinical workload, workforce constraints, and unequal institutional resources likely contribute to these disparities. Service demands often limit time for mentorship and academic activities, while differences between university and non-university centers affect access to research and subspecialty exposure. Without clearly implemented standards for protected learning and mentorship, training experiences may remain uneven across institutions.

For interested oncologists, we suggest creating clinician-scientist tracks or scholarships to encourage PhD enrolment and post-doctoral research. Given that 19% of respondents to our survey express a clear desire for research mentorship, mentoring in research—such as matching trainees with experts—should be improved. In order to integrate academic development into early careers, institutions must also acknowledge research as an integral part of the job. This can be achieved by reducing clinical load for those actively involved in projects or by scheduling dedicated research time into work schedules. Engaging in meaningful research and ongoing learning is known to prevent burnout by offering a sense of accomplishment beyond routine clinical work [3, 19]. These actions could not only boost research engagement but also improve job satisfaction.

### Professional development initiatives

According to our survey, young oncologists in Romania show a strong interest in advancing their careers through training and mobility, yet there is a clear gap between aspirations and outcomes. While over 80% expressed interest in applying for international scholarships or domestic exchange programs, only 16% have actually completed training abroad, highlighting significant barriers such as limited funding, lack of institutional partnerships, personal constraints, and concerns about job security upon return. Similar patterns are observed across Europe, where demand for fellowships consistently exceeds availability [3]. Notably, residents and early-career specialists expressed the highest desire for mobility, and 85% of respondents are already members of the European Society for Medical Oncology (ESMO), reflecting strong engagement with international opportunities. However, only half considered SNOMR’s professional development support sufficient, pointing to room for improvement at the national level.

To bridge this gap, SNOMR, universities, and hospitals could expand collaborations with organizations such as ESMO and ESO, develop exchange programs, and ensure adequate financial support. Clear policies that guarantee recognition of fellowship experience and job stability upon return would also reduce barriers. While international training can equip oncologists with specialized expertise and foster innovation, it also carries the risk of “brain drain” if domestic career opportunities remain limited. Strengthening research support, mentorship, and career pathways within Romania is therefore critical for retaining talent. Encouragingly, SNOMR has recently launched a fellowship programme in collaboration with the European School of Oncology to address these needs. By integrating international exposure with national career development, Romania can modernize its oncology workforce and improve patient outcomes. Continued dialogue between policymakers, academic institutions, and young oncologists will be essential to transform the survey’s findings into sustainable reforms [25].

To our knowledge, no neighboring countries have conducted a comparable analysis in a similar population of respondents. The closest references are the national survey performed in Spain and the broader European survey coordinated by the European Society for Medical Oncology (ESMO), which assessed young oncologists across multiple countries rather than within a single national context.

### Limitations of the study

This study is subject to important methodological limitations. Participation was voluntary and restricted to SNOMR members. The 37% response rate raises concerns regarding representativeness, particularly given the absence of non-respondent analysis. Although the vast majority of Romanian medical oncologists are SNOMR members given the relatively small national workforce, thereby reducing but not eliminating selection bias, our sample is still limited to this group, and the findings should nonetheless be generalized to all early-career oncologists in Romania with appropriate caution.

The extent to which our findings can be applied to all early-career medical oncologists in Romania may have been limited by the voluntary nature of the participation correlated with the response rate, which may have resulted in an overrepresentation of respondents who are affiliated with academic institutions and who are more motivated, engaged, or interested in the survey topic. We recognize that an additional limitation of this study may be the potential underrepresentation of oncologists practicing in non-tertiary or regional healthcare settings.

Furthermore, the descriptive, cross-sectional design, without statistical testing or adjustment for confounders such as institution type, region, or training level, limits the ability to identify significant associations or infer causality.

Nonetheless, these findings provide the first nationwide overview of the professional challenges and aspirations of young oncologists in Romania and offer valuable direction for future workforce development policies.

## Conclusions

Young oncologists in Romania face persistent challenges related to job stability, mentorship, research involvement, and academic development. While there is strong enthusiasm for national and international training opportunities, access remains limited. Insufficient institutional support and lack of protected research time contribute to burnout and restrict career advancement. To build a competent and motivated oncology workforce capable of addressing the country’s growing cancer burden, hospitals, universities, and policymakers must implement coherent strategies that ensure job stability, structured mentorship, dedicated research opportunities, and international mobility. Encouragingly, the Romanian National Society of Medical Oncology has already taken important first steps, launching mentorship and fellowship programs designed to support young oncologists and foster professional growth.

## Supplementary Information

Supplementary Material 1.

Supplementary Material 2.

## Data Availability

The datasets are available from the corresponding author upon request.

## References

[1] Popescu RA, Schafer R, Califano R, Eckert R, Coleman R, Douillard JY, et al. The current and future role of the medical oncologist in the professional care for cancer patients: a position paper by the European Society for Medical Oncology (ESMO). Ann Oncol. 2014;25(1):9–15. 10.1093/annonc/mdt522. 24335854 10.1093/annonc/mdt522

[2] Morgan G, Lambertini M, Kourie HR, Amaral T, Argiles G, Banerjee S, et al. Career opportunities and benefits for young oncologists in the European Society for Medical Oncology (ESMO). ESMO Open. 2016;1(6):e000107. 10.1136/esmoopen-2016-000107. 28255451 10.1136/esmoopen-2016-000107 PMC5174792

[3] Banerjee S, Califano R, Corral J, de Azambuja E, De Mattos-Arruda L, Guarneri V, et al. Professional burnout in European young oncologists: results of the European Society for Medical Oncology (ESMO) Young Oncologists Committee Burnout Survey. Ann Oncol. 2017;28(7):1590–6. 10.1093/annonc/mdx196. 28449049 10.1093/annonc/mdx196 PMC5834057

[4] Romaniuk P, Szromek AR. The evolution of the health system outcomes in Central and Eastern Europe and their association with social, economic and political factors: an analysis of 25 years of transition. BMC Health Serv Res. 2016;16:95. 10.1186/s12913-016-1344-3. 26988369 10.1186/s12913-016-1344-3 PMC4794902

[5] Mazeikaite G, O’Donoghue C, Sologon DM. What drives cross-country health inequality in the EU? Unpacking the role of socio-economic factors. Soc Indic Res. 2021;155(1):117–55. 10.1007/s11205-020-02587-2.

[6] OECD. EU Country Cancer Profile: Romania 2025. Paris: OECD. 2025. Available from: https://www.oecd.org/content/dam/oecd/en/publications/reports/2025/02/eu-country-cancer-profile-romania-2025_ef833241/8474a271-en.pdf. (accessed 24 Sep 2025).

[7] Ministry of Health Romania. Order no. 1225/2011 amending Annex no. 3 to Order no. 1141/1386/2007 on residency training. 2011. Available from: https://legislatie.just.ro/Public/DetaliiDocument/130649. (accessed 10 Sep 2025).

[8] International Agency for Research on Cancer. GLOBOCAN 2022 — Fact Sheet: Romania. Lyon: Global Cancer Observatory. 2022. Available from: https://gco.iarc.who.int/media/globocan/factsheets/populations/642-romania-fact-sheet.pdf. (accessed 10 Sep 2025).

[9] Ministry of Health Romania. Residency examination – November 21, 2010: available positions for medical oncology. 2010. Available from: https://rezidentiat.ms.ro/21112010/locuri_21112010.pdf. (accessed 10 Sep 2025).

[10] Ministry of Health Romania. Residency examination – November 15, 2015: available positions for medical oncology. 2015. Available from: https://rezidentiat.ms.ro/20151115/20151115-locuri.pdf. (accessed 10 Sep 2025).

[11] Ministry of Health Romania. Residency examination – November 19, 2023: available positions for medical oncology. 2023. Available from: https://rezidentiat.ms.ro/20231119/20231119-locuri.pdf. (accessed 10 Sep 2025).

[12] Ministry of Health Romania. Annual activity report 2024. Bucharest: Ministry of Health. 2024. Available from: https://ms.ro/media/documents/Raport_de_activitate_pentru_anul_2024.pdf. (accessed 10 Sep 2025).

[13] Romanian Society of Medical Oncology (SNOMR). Home page. 2025. Available from: https://snomr.ro/main/page/home.php. (accessed 9 Nov 2025).

[14] Seruga B, Sullivan R, Fundytus A, Hopman WM, Ocana A, Joffe J, et al. Medical oncology workload in Europe: one continent, several worlds. Clin Oncol (R Coll Radiol). 2020;32(1):e19–26. 10.1016/j.clon.2019.06.017. 31324474 10.1016/j.clon.2019.06.017

[15] El Bairi K, Al Jarroudi O, Afqir S. Practical tools and guidelines for young oncologists from resource-limited settings to publish excellence and advance their career. JCO Glob Oncol. 2021;7:1668–81. 10.1200/GO.21.00310. 34910583 10.1200/GO.21.00310 PMC8691496

[16] Lim KHJ, Westphalen CB, Berghoff AS, Cardone C, Connolly EA, Guven DC, et al. Young oncologists’ perspective on the role and future of the clinician-scientist in oncology. ESMO Open. 2023;8(5):101625. 10.1016/j.esmoop.2023.101625. 37659290 10.1016/j.esmoop.2023.101625 PMC10480053

[17] Martinez DAS, Quilez-Cutillas A, Jimenez-Labaig P, Sesma A, Tarazona N, Pacheco-Barcia V, et al. Current professional standing of young medical oncologists in Spain: a nationwide survey by the Spanish Society of Medical Oncology + MIR section. Clin Transl Oncol. 2023;25(3):796–802. 10.1007/s12094-022-02989-3. 36418642 10.1007/s12094-022-02989-3 PMC9685015

[18] Hlubocky FJ, Back AL, Shanafelt TD. Addressing burnout in oncology: why cancer care clinicians are at risk, what individuals can do, and how organizations can respond. Am Soc Clin Oncol Educ Book. 2016;35:271–9. 10.1200/EDBK_156120. 27249706 10.1200/EDBK_156120

[19] Copur MS. Burnout in oncology. Oncology (Williston Park)*.* 2019;33(11). 31769864

[20] Ma S, Huang Y, Yang Y, Ma Y, Zhou T, Zhao H, et al. Prevalence of burnout and career satisfaction among oncologists in China: a national survey. Oncologist. 2019;24(7):e480–9. 10.1634/theoncologist.2018-0249. 30568022 10.1634/theoncologist.2018-0249 PMC6656451

[21] ESMO Task Force. Medical Oncology Status in Europe Survey (MOSES Study). 2008. Available from: https://www.esmo.org/content/download/8358/170037/1/2008-ESMO-MOSES-PhaseIII.pdf. (accessed 10 Sep 2025).

[22] Horn L, Koehler E, Gilbert J, Johnson DH. Factors associated with the career choices of hematology and medical oncology fellows trained at academic institutions in the United States. J Clin Oncol. 2011;29(29):3932–8. 10.1200/JCO.2011.35.8663. 21911716 10.1200/JCO.2011.35.8663 PMC3189092

[23] Cammarota A, Siebenhüner AR, Olungu C, Szturz P, Güven DC, Puccini A, et al. Research training, barriers, and career development needs of early-career investigators in oncology: an EORTC survey-based study. ESMO Gastrointest Oncol. 2025;9:100208. 10.1016/j.esmogo.2025.100208. 41647971 10.1016/j.esmogo.2025.100208 PMC12836613

[24] Medisauskaite A, Kamau C. Prevalence of oncologists in distress: systematic review and meta-analysis. Psychooncology. 2017;26(11):1732–40. 10.1002/pon.4382. 28116833 10.1002/pon.4382

[25] Shanafelt TD, Gradishar WJ, Kosty M, Satele D, Chew H, Horn L, et al. Burnout and career satisfaction among US oncologists. J Clin Oncol. 2014;32(7):678–86. 10.1200/JCO.2013.51.8480. 24470006 10.1200/JCO.2013.51.8480 PMC3927737

[26] Vasileva-Slaveva M, Morales-Espinosa D, Puccini A, Meissner M, Milic M, Lamberti G, et al. Tackling hurdles in front of young clinical investigators in oncology – results from an international survey. Eur J Surg Oncol. 2024;50(6):108031. 10.1016/j.ejso.2024.108031. 38552416 10.1016/j.ejso.2024.108031

