# Supplementary material for: Career challenges of young oncologists in Romania: a nationwide survey
Source: BMC Med Educ. 2026 Mar 20;26:690. doi: 10.1186/s12909-026-09006-z (PMC13126845; doi:10.1186/s12909-026-09006-z)
Supplement: Supplementary file 1 — Supplementary Material 1. [file 12909_2026_9006_MOESM1_ESM.pdf]

Survey Questionnaire (English Version)

1. What is your affiliation?
Municipal Hospital
Private Hospital
University Hospital
Oncology Institute

2. What is your professional level?
Resident doctor
Specialist doctor
Senior doctor

3. If you are a resident physician, what year of residency are you in?
1
2
3
4
5

4. What region do you work in?
Transylvania
Moldova
Dobrogea
Oltenia
Crișana
Banat
Maramureș
Bucovina
Muntenia

5. What is your current position?
Resident doctor
Specialist
Senior doctor
Without contract

6. What type of employment contract do you have?
Permanent contract
Fixed-term contract

7. Your current professional activity includes:
Clinical work
Research
Pharmaceutical industry
Health management

Teaching
Non-oncology field

8. How many hours are specified in your contract?
40 hours/week (8 per day)
37.5 hours/week
35 hours/week (7 per day)
Less than 35 hours/week

9. How many extra hours do you work beyond contract hours?
1 hour/day
2 hours/day
3 hours/day
4 hours/day
More than 4 hours/day
I do not work extra hours

10. Are extra hours paid?
Yes
No

11. What is the main purpose of your contract?
Clinical activity
Research
Teaching

12. Do you believe your working hours align with the main objective of your contract?
Yes
No

13. Were you offered the possibility of a permanent job in your current department?
Yes
No

14. How did you obtain your current position?
Jobs available from ads on the website
Interview and CV presentation with the head of department
Scholarships
Position obtained through residency competition
Position obtained through competition with an ad published on the website of the Ministry of Health/employing hospita

15. How concerned are you about job stability (defined as remaining in the same job for more than 1 year)?
Scale: 1 = least concerned → 10 = most concerned

16. To your knowledge, which of the following have occurred?

Public job offer in your specialty
Job consolidation process
No actions taken to secure stable employment
I don’t know

17. How many contracts have you signed in the past 2 years?
None
1
2
3
4
5 or more

18. Have you considered alternative career paths besides clinical activity?
Yes
No
Yes, pharmaceutical industry
Yes, research only
Yes, teaching only

19. After training, do you plan (or have you planned) to move abroad?
Yes
No

20. If yes, where?
European Union
Outside European Union

21. What is the reason?
Higher income
Professional development
Personal reasons
Other: _______

22. What is your opinion about employment conditions in Romanian oncology compared to other countries?
Better
Similar
Worse
Don’t know

23. If you're a young specialist, do you feel uncertain about your situation?
Yes
No
I’m not a specialist yet

24. Have you experienced burnout during residency/specialization or in the past 5 years?
Yes
No

25. If yes, did you seek treatment?
Yes
No

26. If yes, how many burnout episodes in the last year?
1
2
3
More than 3

27–31. In the past 2 weeks, how often have you felt the following:
Cheerful and in good spirits
Calm and relaxed
Active and energetic
Well-rested upon waking
Daily life full of interesting things

Options:
All the time
Most of the time
More than half the time
Less than half the time
Rarely
Not at all

32. Do you feel your current job offers a good work-life balance?
Yes
No

33. Do you believe your job expectations were met after residency?
Yes
No

34. If no, what are the reasons?
Uncompetitive salary
Lack of career advancement
Lack of protected time for patient care/research/education

35. Are you happy with your current job?
Yes

No

36. If no, why not?
Workplace atmosphere
Workload too high
Lack of facilities
Boss/Manager
Salary

37. Do you have a mentor at your institution?
Yes
No

38. If yes, who?
Senior resident
Chief resident
Specialist / Senior doctor
Professor
Other: _______

39. Who has helped or supervised your professional growth as an oncologist?
Specialist / Senior doctor
Mentor (advised/guided you professionally)
Sponsor (provided an employment opportunity)

40. Would you be interested in a mentorship program by SNOMR?
Yes
No

41. Would you be interested in ESMO/ESO mentorship program?
Yes
No

42. What are your expectations/needs from a mentor or mentorship program?
(Open-ended)

43. Are you currently pursuing a PhD?
Yes
No

44. Have you completed a PhD?
Yes
No

45. Are you involved in clinical trials at your institution?

Yes
No

46. Do you have academic/educational responsibilities?
Yes
No

47. How much of your time is allocated to research?
None
<25%
25–50%
50%

48. Do you do research during or outside working hours?
During work hours
Outside work hours
Both

49. Have you obtained funding for your research?
Yes
No

50. Have you published a scientific article in the past year?
Yes
No

51. If not, have you published in the past 5 years?
Yes
No

52. Do you think oncology training in Romania supports meaningful career development?
Yes
No

53. Are there training courses available at your institution?
Yes
No

54. Do you think your institution invests enough in your professional development?
Yes
No

55. If not, why?
Lack of funding

Lack of support
Lack of interest

56. Do you have the opportunity to focus on a subspecialty in medical oncology (tumor-specific)?
Yes
No

57. How many hours per day do you dedicate to career development?
None
1 hour
2 hours
3 hours
4 hours

58. Would you consider an exchange program within Romania?
Yes
No

59. Are you considering a fellowship abroad?
Yes
No

60. Have you completed a fellowship abroad?
Yes
No

61. How many oncology articles do you read per month?
None
1
2–5
5–10
10

62. Are you currently an ESMO member?
Yes
No

63. Do you think SNOMR offers enough development opportunities?
Yes
No
