# Supplementary material for: Career challenges of young oncologists in Romania: a nationwide survey
Source: BMC Med Educ. 2026 Mar 20;26:690. doi: 10.1186/s12909-026-09006-z (PMC13126845; doi:10.1186/s12909-026-09006-z)
Supplement: Supplementary file 2 — Supplementary Material 2. [file 12909_2026_9006_MOESM2_ESM.pdf]

Educational Indicator	Residents (n=91)	Specialists (n=63)	Consultants (n=15)
Academic/Educational Responsibilities (Q46) – Yes	15%	23%	33%
Clinical Trial Involvement (Q45) – Yes	20%	44%	47%
No Research Activity (Q47)	52%	42%	27%
Currently Pursuing PhD (Q43)	20%	19%	20%
Completed PhD (Q44)	0%	19%	33%
Published Scientific Article in Past Year (Q50) – Yes	18%	34%	47%
